# Use of Active Salmon-Lecithin Nanoliposomes to Increase Polyunsaturated Fatty Acid Bioavailability in Cortical Neurons and Mice

**DOI:** 10.3390/ijms222111859

**Published:** 2021-11-01

**Authors:** Elodie Passeri, Kamil Elkhoury, Maria Camila Jiménez Garavito, Frédéric Desor, Marion Huguet, Claire Soligot-Hognon, Michel Linder, Catherine Malaplate, Frances T. Yen, Elmira Arab-Tehrany

**Affiliations:** 1 LIBio Laboratory, University of Lorraine, 54505 Vandoeuvre-lès-Nancy, France; elodie.passeri@univ-lorraine.fr (E.P.); kamil.elkhoury@univ-lorraine.fr (K.E.); camijimenez9426@hotmail.com (M.C.J.G.); michel.linder@univ-lorraine.fr (M.L.); 2 UR AFPA Laboratory, Qualivie Team, University of Lorraine, 54505 Vandoeuvre-lès-Nancy, France; frederic.desor@univ-lorraine.fr (F.D.); marion.cizo@univ-lorraine.fr (M.H.); claire.soligot@univ-lorraine.fr (C.S.-H.); catherine.malaplate-armand@univ-lorraine.fr (C.M.)

**Keywords:** polyunsaturated fatty acids, omega-3 fatty acids, nanoliposomes, bioavailability, brain, cortical neurons, mouse

## Abstract

Omega-3 polyunsaturated fatty acids (n-3 PUFAs) play an important role in the development, maintenance, and function of the brain. Dietary supplementation of n-3 PUFAs in neurological diseases has been a subject of particular interest in preventing cognitive deficits, and particularly in age-related neurodegeneration. Developing strategies for the efficient delivery of these lipids to the brain has presented a challenge in recent years. We recently reported the preparation of n-3 PUFA-rich nanoliposomes (NLs) from salmon lecithin, and demonstrated their neurotrophic effects in rat embryo cortical neurons. The objective of this study was to assess the ability of these NLs to deliver PUFAs in cellulo and in vivo (in mice). NLs were prepared using salmon lecithin rich in n-3 PUFAs (29.13%), and characterized with an average size of 107.90 ± 0.35 nm, a polydispersity index of 0.25 ± 0.01, and a negative particle-surface electrical charge (−50.4 ± 0.2 mV). Incubation of rat embryo cortical neurons with NLs led to a significant increase in docosahexaenoic acid (DHA) (51.5%, *p* < 0.01), as well as palmitic acid, and a small decrease in oleic acid after 72 h (12.2%, *p* < 0.05). Twenty mice on a standard diet received oral administration of NLs (12 mg/mouse/day; 5 days per week) for 8 weeks. Fatty acid profiles obtained via gas chromatography revealed significant increases in cortical levels of saturated, monounsaturated, and n-3 (docosahexaenoic acid,) and n-6 (docosapentaenoic acid and arachidonic acid) PUFAs. This was not the case for the hippocampus or in the liver. There were no effects on plasma lipid levels, and daily monitoring confirmed NL biocompatibility. These results demonstrate that NLs can be used for delivery of PUFAs to the brain. This study opens new research possibilities in the development of preventive as well as therapeutic strategies for age-related neurodegeneration.

## 1. Introduction

Lipids are a heterogeneous group of hydrophobic or amphiphilic molecules, and play an important role in all cell types in mammals. They contribute to the production and storage of energy; serve as building blocks of cell membranes, thereby influencing their fluidity and function; and participate in biological processes, including gene transcription, and the regulation of vital metabolic pathways and physiological responses [1,2,3].

The brain is among the organs that are richest in lipids, along with the adipose tissue. The brain contains high levels of long-chain polyunsaturated fatty acids (PUFAs), which represent approximately 25–30% of the total fatty acids in the brain, and 20% of the brain’s dry weight, including mainly docosahexaenoic acid (DHA, 22:6n-3), arachidonic acid (AA, 20:4n-6) and, to a lesser extent, eicosapentaenoic acid (EPA, 20:5n-3) [4,5]. DHA and AA represent 13–22% and 5–11% of brain phospholipids, respectively [6]. Linoleic (LA C18:2n-6) and α-linolenic (ALA C18:3n-3) acids are precursors required for the synthesis of AA (LA), EPA, and DHA (ALA), via desaturation and elongation. LA and ALA are both essential fatty acids that are obtained through diet, and are present only at low concentrations in the brain [2,7].

PUFAs are important in brain development, integrity, and function, and a balanced n-6/n-3 ratio in the diet is particularly important for health and wellbeing with age [8]. Indeed, a high n-6/n-3 PUFA ratio is associated with increased risk of metabolic syndrome, cardiovascular and inflammatory diseases [9], and neurodegenerative diseases such as Alzheimer’s disease (AD) [10,11]. Studies show that n-3 PUFAs as dietary supplements are useful in reducing the risk of a number of disorders, including metabolic, cardiovascular, inflammatory, autoimmune, and neurological diseases [8,9].

Investigations have focused particularly on DHA, which is also the most abundant n-3 PUFA in the brain, representing 90% of the n-3 PUFAs in this tissue [12,13,14]. DHA is found at high levels in the nervous system—particularly in membrane photoreceptors and synaptic membranes; it is stored mainly in membrane phospholipids of phosphatidylethanolamine (PE) and phosphatidylserine (PS), with smaller amounts in phosphatidylcholine (PC). Studies on the neuroprotective effects of DHA, including those on synaptic plasticity and neurogenesis, revealed that this n-3 PUFA affects numerous cellular functions and physiological processes, including gene expression, membrane fluidity, lipid raft function, neurotransmitter release, transmembrane receptor functions, signal transduction, myelination, neuronal differentiation, neuronal growth, and inflammation [15,16,17,18]. DHA has been shown to play an important role in brain processes including memory, vision, and corneal nerve regeneration [19,20,21]. Several in vitro and in vivo studies have reported that n-3 PUFAs can improve cognitive functions, and may play a role in the prevention of cognitive decline [22]. Clinical studies using DHA supplements in the elderly or diets rich in fish-derived n-3 PUFAs suggest that this n-3 PUFA may help reduce the risk of mild cognitive decline [23,24,25]. We recently reported that dietary DHA supplementation can be used as an adjuvant in aged mice to preserve neuronal membrane integrity, thus allowing membrane receptors such as those for the ciliary neurotrophic factor (CNTF) to react appropriately to anti-AD agents such as CNTF [26]. Therefore, DHA supplementation in the diet could prevent age-related neuronal membrane changes and associated dysfunction.

Changes in the lipid composition of neuronal membranes represent a risk factor for the development of neurological diseases [13,27] and affective disorders [28,29,30]. In addition, it has been reported that the total volume of gray matter decreases with age, along with a parallel decrease in DHA levels [11], since DHA is found at particularly high levels in gray matter [12]. This demonstrates the need for supplementation of n-3 PUFAs to the central nervous system for the prevention of neuronal dysfunctions, thus reducing the risk of age-related neurodegeneration and cognitive deficits [31]. N-3 PUFA supplementation in elderly individuals without neurological diseases [32] has shown promising preventive potential for maintaining cognitive functions and, in the early stages of AD, for delaying cognitive decline [27]. However, the results are more moderate for advanced neurodegenerative disorders, where it appears that only people with mild cognitive decline and who are not at risk of developing AD benefit from improved cognitive performance. Furthermore, the effects observed are generally short-lived [31].

However, to achieve balanced n-6 and n-3 PUFA levels requires developing a means of targeted delivery of these lipids to the brain that is able to overcome the blood–brain barrier (BBB), which limits access to the brain.

New techniques to facilitate the access of therapeutic molecules to the brain are currently being developed, among which are nanoliposomes (NLs), considered to be among the most promising and effective drug delivery systems [33,34]. Liposomes were first described by A.D. Bangham in the 1960s [35], and have been studied extensively for drug and nutrient delivery. Liposomes are spherical and self-closed structures formed by one or several concentric lipid bilayers, with an aqueous phase inside and between the lipid bilayers. The size of a liposome can range from 50 nm to several µm in diameter, depending on lipid composition and preparation methodology [36]. Lipid particles can be prepared using different classes of lipids, but phospholipids are the major components of liposome membranes, by virtue of their amphiphilic nature [37,38]. Liposomes can be considered to be an excellent drug delivery system, by virtue of their properties being similar to those of cell membranes [39]. They have the advantage of being relatively straightforward to produce, with good biocompatibility and biodegradability, low toxicity, drug-targeted delivery, and controlled drug release [33,34,38,39]. Drug delivery to the brain is a very complex phenomenon, and remains a challenge in the development of therapeutic strategies. Drugs targeting PUFAs may lead to novel therapeutic and nutraceutical approaches [40] to the prevention and treatment of brain disorders. Investigation is ongoing to test new techniques to facilitate the access of therapeutic molecules to the brain, including soft nanoparticles and, more specifically, NLs. Soft nanoparticles can cross the BBB via passive or active targeting techniques, and can deliver the appropriate amount of therapeutic agents to the brain [41].

In our laboratory, we developed a method for the preparation of n-3 PUFA-rich NLs from salmon lecithin obtained via green extraction from salmon head byproducts. Studies using primary cultures of rat cortical neurons demonstrated beneficial effects of the NLs on neuronal growth and development, along with synaptogenesis [42,43,44] and, thus, potential neuroprotective effects of these salmon-lecithin-derived NLs.

This led us to question whether these n-3 PUFA-rich NLs could represent a means of delivery of PUFAs to the brain. The objective of this study was to investigate the NL-mediated delivery of PUFAs in a neuronal cell culture model, and in vivo using mice as an animal model.

## 2. Results

### 2.1. Fatty Acid Composition of Salmon Lecithin

Salmon lecithin was prepared from salmon head byproducts as described in the Section 4. Main FA composition analysis via gas chromatography (Table 1) showed that 41.35% of total FAs in salmon lecithin were PUFAs, of which n-3 PUFAs were the most abundant fatty acids, at 29.13% of total FAs, with an n-6/n-3 ratio of 0.42. DHA and EPA levels were 18.04% and 7.55%, respectively. Salmon lecithin also contained 31.09% MUFAs—primarily oleic acid (C18:1n-9, 26.16%)—and 27.56% SFAs, particularly palmitic acid (C16:0, 20.08%). Salmon lecithin contained relatively equivalent amounts of PUFAs, MUFAs, and SFAs, similar to what was reported previously for lecithin derived from salmon head byproducts [45].

### 2.2. Lipid Classes of Salmon Lecithin

The lipid classes of salmon lecithin were determined via thin-layer chromatography. The results showed that this lecithin was composed of 67.65 ± 0.90% phospholipids, 31.20 ± 0.40% triglycerides, and 1.15 ± 0.10% total cholesterol (*n* = 10). Phosphatidylcholine (PC) was found to be the main class of phospholipid, representing 42% of the total phospholipids. Therefore, the salmon lecithin used for NLs in this study was composed primarily of phospholipids, and also contained neutral lipids as well as low levels of total cholesterol.

### 2.3. Physicochemical Characterization of Nanoliposomes

**Figure 1:**
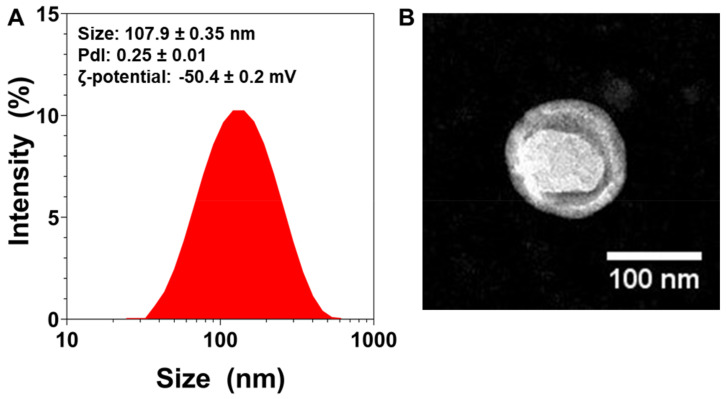
Physicochemical characterization of nanoliposomes (NL): (**A**) Size distribution measurements of NLs measured via dynamic light scattering, with average size, polydispersity index (PDI), and zeta-potential values (*n* = 3). (**B**) TEM images of NLs.

Immediately after their preparation, the NLs’ average particle size was measured. The mean particle size was found to be 107.90 ± 0.35 nm (*n* = 3) (Figure 1A). The NLs’ size was further confirmed via TEM (Figure 1B), which also revealed their spherical morphology.

The polydispersity index (PDI) is a dimensionless measure of particle size distribution, and is also measured via dynamic light scattering (DLS) [46]. Figure 1A shows that the NLs’ PDI was 0.25, which indicates that the particles had a controlled size distribution and a narrow dispersity.

To characterize the particles’ surface electrical charge, ζ-potential measurements were conducted [47]. The NLs’ ζ-potential was found to be very negative (~−50 mV, Figure 1A), which can lead to more stable colloidal dispersions, since the greater the ζ-potential magnitude, the greater the repulsion between particles [48].

### 2.4. Effect of Nanoliposomes on the Fatty Acid Composition of Primary Cultures of Embryo Cortical Neurons

To study the potential bioavailability of NLs, we first sought to determine whether NL treatment of neurons could lead to changes in FA composition. Primary cultures of embryo cortical neurons were incubated in the absence (C) or presence (NL) of 10 µg/mL NLs. These concentrations were previously shown to lead to neurotrophic effects in this cell culture model [42]. Cells were recovered after 24 h, 48 h, and 72 h of incubation for lipid extraction and FA composition analysis via gas chromatography (Table 2).

**Figure 2:**
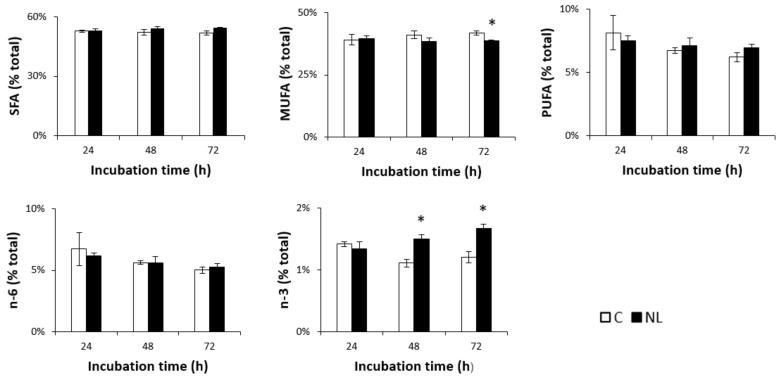
SFA, MUFA, PUFA, n-3, and n-6 profiles (% total) in primary cultures of rat embryo cortical neurons treated with NLs and in the control group depending on incubation time. Cells were incubated at 37 °C in the absence (C) or presence (NL) of 10 µg/mL NLs. Fatty acid composition was analyzed via gas chromatography after 24 h, 48 h, and 72 h of incubation. The values are expressed as the mean ± SD of % total fatty acids. SFAs: saturated fatty acids; MUFAs: monounsaturated fatty acids; PUFAs: polyunsaturated fatty acids; n-6: n-6 fatty acids; n-3: n-3 fatty acids. Statistical differences between groups for each fatty acid are shown within each timepoint (* *p* < 0.01).

Lipid analysis showed that FA composition in neurons was in the range of 50–55% SFAs, 40% MUFAs, and 5–10% PUFAs. A small but significant decrease in MUFA levels was observed in NL-treated cells after 72 h. While n-6 PUFA levels did not change over time in either the control or the NL-treated cells, n-3 PUFA levels increased significantly at 48 h (+35.1%, *p* < 0.05) in cells treated with NLs, compared to controls (Figure 2). This did not significantly increase after 72 h, suggesting that maximal n-3 PUFA enrichment of the cortical neurons was observed after 48 h.

Statistical analysis of the relative changes in individual FAs revealed significantly higher levels of DHA as a % of total FAs in NL-treated cortical cells compared to controls, but only after 72 h of incubation. Upon closer examination, the % of DHA levels in NL-treated neurons actually remained relatively unchanged between the three different timepoints, while those in control cells decreased over time. This would suggest that the cortical neurons in NL-enriched media were able to maintain DHA levels during this extended time, unlike the control cells. An increase in % EPA was observed in both control and NL-treated cells; this was significantly higher in NL-treated cells at 48 h. Since DHA–EPA interconversion does occur, this may have contributed to the decreased DHA in control cells. Although there was no significant change in SFAs as a group, a small but significant increase in palmitic acid (C16:0) was observed after 72 h of incubation with NLs when compared to controls (Table 2). C16:0 is a common FA; moreover, this increase might reflect the incorporation of this FA from NLs, in which 20% of total FAs are C16:0. Lipid analysis revealed slightly lower levels of MUFAs after 72 h of incubation with NLs, due primarily to C18:1n-9, which is significantly decreased in NL-treated cells.

### 2.5. Effect of 8 Weeks of Nanoliposome Treatment on the Fatty Acid Compositions of the Brain and Liver in Mice

#### 2.5.1. Weight, Food Intake, and Plasma Lipid Levels

In view of NL-mediated FA delivery to cultured neurons, our next objective was to determine NL bioavailability in vivo, using mice as an animal model. C57BL6J mice received NLs via gavage for 5 days per week for 8 weeks. Weight and food intake were monitored biweekly throughout the study. No significant changes in body mass or food intake were observed during the experimental period (data not shown).

Plasma total cholesterol (TC) and triglyceride (TG) levels were determined at T0 and after 8 weeks of NL treatment. TC levels were 100.80 ± 11.75 mg/dL and 102.04 ± 15.32 mg/dL in the C and NL groups, respectively; plasma TG levels were 28.17 ± 18.40 mg/dL and 26.90 ± 25.22 mg/dL in the C and NL groups, respectively. There was no statistically significant difference for either parameter, suggesting that NL treatment via gavage did not lead to any effects on plasma lipid levels. Therefore, the small amounts of TG and cholesterol in the NLs did not lead to hyperlipidemia. The mice were monitored daily for any behavioral changes and possible signs of discomfort; none were observed during the experimental period, showing adequate tolerance of the NL administration.

#### 2.5.2. Tissue Samples

**Figure 3:**
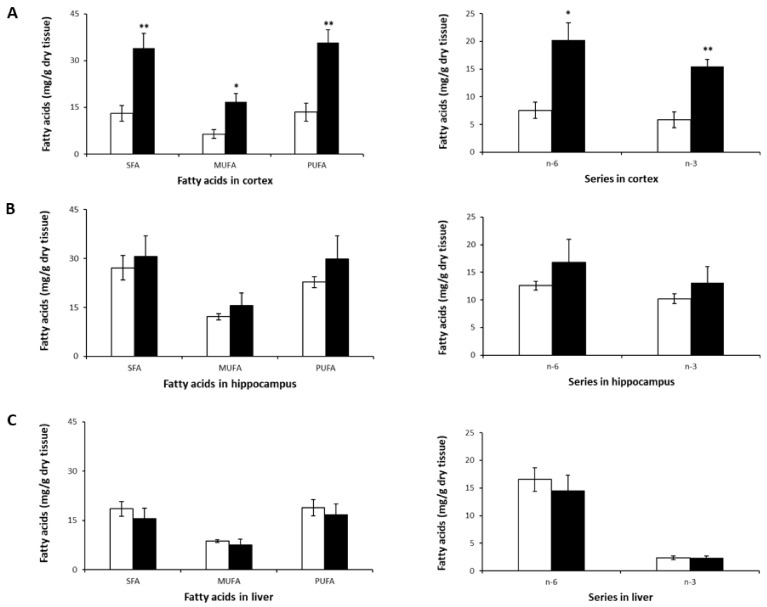
SFA, MUFA, PUFA, n-3, and n-6 profiles in murine cortex (**A**), hippocampus (**B**), and liver (**C**) after 8-week NL treatment. Results are shown for the control (open bars) and NL (closed bars) groups. Results are expressed as mean ± SD (*n* = 5/group). Statistical differences comparing the control and NL groups are shown for each tissue (* *p* < 0.05, ** *p* < 0.01).

After 8 weeks, the animals were euthanized, and tissues including the liver, cortex, and hippocampus were excised for lipid extraction, followed by gas chromatography to obtain fatty acid profiles (Table 3 and Figure 3).

The most striking observation was the significant increase in SFA, MUFA, and PUFA levels (both n-6 and n-3) in the cortex (Table 3, Figure 3A). A tendency towards an increase in these FA classes was observed in the hippocampus (Figure 3B), but none reached statistical significance. In the liver, LA (C18:2n-6) was higher compared to its levels in the brain, which is consistent with previous studies on mice [49,50]. However, no significant differences were observed in SFA, MUFA or PUFA levels (Figure 3C) following treatment with NLs in mice.

The increased PUFA levels in the cortices of mice in the NL group was due to higher levels of not only n-3 DHA, but also n-6 AA, and n-6 docosapentaenoic acid (DPA)—a metabolite of LA—compared to controls. There was an almost threefold increase in cortical DHA and n-6 DPA content (Table 3).

Cortices from mice that received NLs also showed significant increases in SFA and MUFA levels compared to the control group, due primarily to higher levels of palmitic (C16:0), stearic (C18:0), and oleic (C18:n-9) acids. This is similar to the increase in SFAs observed in cortical neurons treated with the same types of NLs. No statistically significant intergroup differences were observed for SFAs or for MUFAs in the hippocampus and the liver (Figure 3B,C).

Despite the significant increases in FA levels in the cortex, no differences in the ratios of n-6/n-3, AA/DHA, PUFA/SFA, or MUFA/SFA were observed (Table 3). This was also the case for the hippocampus and the liver.

Analysis of the total amount of lipids in each tissue over time revealed a significant increase in the quantity of cortical lipids at the end of NL treatment, as compared to controls, which may have been due to the higher levels of FAs found in this tissue compared to the control group (Table 4). Conversely, in the liver, although the amount of lipids increased after the 8-week period, the total lipid amounts were significantly lower in the mice given NLs as compared to the control group.

## 3. Discussion

The objective of this study was to assess the ability of these NLs to deliver PUFAs in cellulo and in vivo (in mice). The NLs were prepared using salmon lecithin rich in n-3 PUFAs similar to the FAs and polar head phospholipid profile found in the human brain. Salmon lecithin is a mixture of lipids composed primarily of polar lipids (phospholipids) and neutral lipids—significant constituents of the CNS.

First, we assessed the effect of NLs on the FA composition of primary cultures of embryo cortical neurons. Treatment of rat embryo cortical neurons with NLs showed that n-3 PUFA levels increased significantly in cells treated with NLs, as compared to controls. More precisely, we observed significantly higher levels of DHA in NL-treated cortical cells as compared to controls. Lipid analysis also revealed slightly lower levels of MUFAs, due primarily to C18:1n-9, which was significantly decreased in NL-treated cells. A similar compensatory effect of lower brain MUFAs due to oleic acid was also observed in mice receiving dietary supplementation of n-3 PUFAs; the authors proposed that this may be due to an increase in n-3 PUFAs, although the molecular mechanisms involved are not clearly understood [6,51]. Taken together, these results demonstrate the delivery and assimilation of FAs from NLs to cortical neurons.

Secondly, we studied the effect of 8 weeks of NL treatment on the FA composition of the brain and liver in mice. The NL formulation did not cause deleterious effects on the weight or lipidemia, nor on the general behavior of treated mice. The most striking observation was the significant increase in SFA, MUFA, and PUFA levels (both n-6 and n-3) in the cortex. A tendency towards an increase in these FA classes was observed in the hippocampus, but none reached statistical significance. No significant differences were observed in the liver in SFA, MUFA, or PUFA levels following treatment with NLs in mice. The lack of significant changes in liver PUFA levels may have been due to the conversion of ALA and LA to DHA, n-6 DPA, and AA for release into the bloodstream as lipoproteins for delivery to the brain [18,52]. The increased PUFA levels in the cortices of mice in the NL group was due to higher levels of not only n-3 DHA, but also n-6 AA and n-6 docosapentaenoic acid (DPA)—a metabolite of LA—compared to controls. Studies have shown that FA composition is brain-region specific, with the cortex in particular showing higher PUFA levels [51,53,54]. In addition, studies indicate that this region may be more responsive to dietary changes in FAs [6,54,55]. EPA levels were found to be among the lowest of PUFAs in the cortex and hippocampus; this is consistent with its metabolism, as it is quickly converted by β-oxidation, elongation, and desaturation into DPA and DHA [1,56]. from the cortices of mice that received NLs also showed significant increases in SFA and MUFA levels compared to the control group. No statistically significant intergroup differences were observed for SFAs or MUFAs in the hippocampus and the liver.

The overall brain FA profile in the NL group was similar to those reported in other studies [6,54,55,57]. Here, higher cortical levels of n-6 DPA, along with AA and DHA, contributed to the significant increase in PUFAs in the NL-treated group. This differs from studies that reported an increase in n-6 DPA in animals receiving a high-carbohydrate diet [55] or an n-3 PUFA-deficient diet [6], leading to the observation that brain DHA levels are inversely related to DPA n-6 levels [18]; both FAs are produced in peroxisomes, then used as substrates in membrane lipid biosynthesis. On the other hand, Green et al. showed that n-6 DPA reduced levels of phospho-tau involved in the pathology of AD, and suggested that a combination of DHA + n-6 DPA may be a beneficial natural therapeutic strategy for AD. N-3 and n-6 PUFAs combined may be more effective than DHA supplementation alone [58]. Despite the significant increases in FA levels in the cortex, no differences in the ratios of n-6/n-3, AA/DHA, PUFA/SFA, or MUFA/SFA were observed; this was also the case for the hippocampus and the liver. Since no significant changes were observed in plasma lipid levels, we can conclude that NL supplementation led to increased levels of fatty acids in the cortex, without modifying lipid homeostasis in either the brain or in the liver.

In this study, we chose to use young mice provided with a standard chow ad libitum during the NL treatment; no n-3 or PUFA deficiency was induced. In other studies, any intergroup difference [55,57] or age difference [6] influenced the effects of increased DHA levels in the treated group, or the effects of low levels of DHA in the brains of the control group. In our study, the significant cortical FA levels in adult mice with normal n-3 levels illustrate the possibility of enriching this brain region with PUFAs. This cerebral structure plays an important role in processing sensory information [59], social cognition [60], high cognitive processes such as working memory, attentional control, reasoning, decision making [61], and category learning and categorization [62]. Analysis of the total amount of lipids in each tissue over time revealed a significant increase in the quantity of cortical lipids at the end of NL treatment, as compared to controls, which may have been due to the higher levels of FAs found in this tissue compared to the control group. Conversely, in the liver, although the amount of lipids increased after the 8-week period, the total lipid levels were significantly lower in the mice given NLs compared to the control group. These results are consistent with experimental and clinical studies showing the lipid-lowering effects of n-3 PUFAs via the regulation of hepatic lipid metabolism [49,50,63]. This formulation of NLs allows brain enrichment with PUFAs without accumulation at the hepatic level. This highlights the targeted bioavailability of NLs for the brain, and the decreased risk of hepatic steatosis that can be associated with diets high in fat.

The results of our study demonstrate for the first time the bioavailability of PUFA NLs in neuron cell culture, and the ability of NLs administered orally to enrich PUFA levels in the brain cortex after 8 weeks. N-3 PUFA enrichment following NL administration was higher in the brain than in the liver, without weight gain or hyperlipidemia, with the highest levels in the cortex as compared to the hippocampus; both regions participate in learning, as well as short- and long-term memory. By virtue of the origin and green extraction methods used to obtain salmon lecithin, these NLs are also low in toxicity, and biocompatible in vivo.

Future studies are necessary in order to determine the evolution of cerebral n3-PUFA enrichment beyond 8 weeks, and to more precisely define the optimal duration of supplementation with n-3 PUFAs to maintain CNS lipid homeostasis. Whether this enrichment by NLs is due to direct delivery of NLs crossing the BBB to the brain, or a result of processing in the liver in the form of phospholipids in lipoproteins, remains to be determined. DHA in the form of lysophospholipids (lysophosphatidylcholine) may be able to cross the BBB [64,65,66,67], which would support the latter hypothesis. However, this is specific to DHA, and may not be the only reason for the observed cortical enrichment of other classes of fatty acids, which are also present in the salmon lecithin NLs.

Our results show that oral administration in vivo of unmodified NLs can lead to specific enrichment of the brain cortex with n-3 PUFAs, while bypassing any excess and potentially toxic accumulation of fatty acids in the liver. Indeed, plasma lipemia remained normal during this study, indicating no increased hepatic lipoprotein production due to any increased influx into this tissue. Similarly, there were no changes in body mass or food intake, suggesting no increased body fat mass. This would suggest that NLs can directly access the brain via a mechanism that remains to be defined.

In order to cross the BBB, there are essentially three potential pathways: passive diffusion through the endothelial membrane, active transport via transfer into the endothelium by transmembrane protein transporters (transcytosis), or receptor-mediated endocytosis [68].

The present study was not designed to analyze unmodified NL transport across the BBB via a passive or an active process. However, it is possible to functionalize liposomes [69], and we would propose that modified salmon lecithin NLs targeting the BBB provide not only the means of the delivery of n-3 PUFAs, but also a vector for targeting drugs to the brain.

We would propose that salmon lecithin NLs can be used as supplements for the delivery of n-3 rich PUFAs to the brain, thereby maintaining neuron function and synaptic plasticity, preventing age-related cognitive deficits, and reducing the risk of neurodegenerative diseases such as AD. This study opens new research possibilities in the development of preventive as well as therapeutic strategies for age-related neurodegeneration.

## 4. Materials and Methods

### 4.1. Lecithin Extraction

Lecithin was extracted and purified from *Salmo salar* (salmon) head byproducts using low-temperature enzymatic hydrolysis without any organic solvent [45,70].

### 4.2. Fatty Acid Composition Analysis via Gas Chromatography

Fatty acid methyl esters (FAMEs) were analyzed as previously described, according to the amount of lipids [71,72]. FAME separation was carried out via gas chromatography (Perichrom, Saulx-lès-Chartreux, France). Injector and detector temperatures were fixed at 250 °C. Column temperature was initially set at 120 °C for 3 min, then it was increased to 180 °C at a rate of 2 °C min^−1^ and kept at 220 °C for 25 min. Standard mixtures (PUFA1 from marine source and PUFA2 from vegetable source, Supelco, Sigma–Aldrich, Bellefonte, PA, USA) and a nonadecanoic acid internal control were used to categorize fatty acids. All runs were performed in triplicate.

### 4.3. Lipid Classes of Salmon Lecithin

Lipidic classes of salmon phospholipids were determined using an Iatroscan (MK-5 TLC-FID, Iatron Laboratories Inc., Tokyo, Japan), as described in detail previously [73]. Two migrations were executed to characterize the proportion of polar and neutral lipid fractions. Area percentages were shown as the average of three repetitions.

### 4.4. Preparation of Nanoliposomes

NLs were prepared as previously described to yield a homogeneous monodisperse solution of nano-sized liposomes. Briefly, salmon lecithin was prepared at a concentration of 2% (*w*/*w*) in distilled water. The solution was capped under N_2_(g) to prevent lipid oxidation, and incubated at room temperature with gentle stirring for 4 h. The mixture was sonicated (Vibra-Cell 75115 Sonicator, 500 Watt, Bioblock Scientific Co) for 4 min (pulse: 1 s on and 1 s off) at 40 kHz at 40% of full power. Aliquots of NLs were stored under N_2_(g) in the dark at 4 °C.

### 4.5. Nanoliposomes Characterization

The average hydrodynamic particle diameter (Hd), polydispersity index (PDI), and ζ-potential of the prepared blank and drug-loaded nanoliposomes were characterized via DLS with a Zetasizer Nano ZS device (Malvern Instruments Ltd., Malvern, UK). Prior to measuring size and ζ-potential, the samples were diluted (1:200) with ultrapure distilled water. Measurements were performed at 25 °C with a fixed scattering angle of 173°, the refractive index (RI) at 1471, and absorbance at 0.01. The measurements were performed in standard capillary electrophoresis cells equipped with gold electrodes (DTS 1070). At least three independent measurements were performed for each condition.

### 4.6. Transmission Electron Microscopy (TEM)

Blank and naringin-loaded nanoliposomes’ structures were observed using transmission electron microscopy (TEM) via a negative staining method, as described by Colas et al. [74]. Briefly, to reduce the concentration of nanoliposomes, samples were diluted with ultrapure distilled water (25-fold). To stain nanoliposomes, equal volumes of the diluted solution and an aqueous solution of ammonium molybdate (2%)—used as a negative staining agent—were mixed. After the staining procedure, samples were kept at room temperature for 3 min, followed by a 5 min incubation on a copper mesh coated with carbon. Finally, samples were observed using a Philips CM20 TEM coupled with an Olympus TEM CCD camera.

### 4.7. Primary Culture of Cortical Neurons

Primary cultures of cortical neurons were prepared as described previously [75], and were performed on the Bioavailability–Bioactivity (Bio-DA) platform. Briefly, pregnant female Wistar rats (day 17 of gestation) were euthanized by gas anesthesia (isoflurane), and the embryos were removed, from which the embryos’ brains were collected and separated into the two demi-hemispheres. Following removal of the meninges, cortices were collected for enzymatic digestion using trypsin/EDTA in MSS medium. After tissue dissociation and centrifugation to remove debris, cells were plated at 10 × 10^4^ cells/cm^2^ in Petri dishes pre-coated with poly-L-ornithine (15 µg/mL, Sigma). The cells were cultured in M2neuronal culture medium composed of serum-free DMEM-F12 medium (Invitrogen, Illkirch, France) containing 0.5 µM insulin, 60 µM putrescine, 30 nM sodium selenite, 100 µM transferrin, 10 nM progesterone, and 0.1% (*w*/*v*) ovalbumin, all purchased from Sigma. The cell cultures were maintained at 35 °C in a humidified 6% CO_2_ atmosphere. Neurons were incubated for up to five days, with day (D) 0 as the day of the preparation of the primary culture. NLs were added to D3 at a concentration of 10 µg/mL; this concentration was demonstrated to be nontoxic to cells [42]. After incubation, cells were recovered by scraping, washed in PBS, and centrifuged. Pellets were suspended in PBS and transferred to glass tubes, followed by lipid extraction using the Folch method, and methylation for fatty acid composition analysis using GC, as described above. Cells were recovered, and lipid extraction was performed after 24 h, 48 h, and 72 h of incubation in the presence of NLs.

### 4.8. In Vivo Study

#### 4.8.1. Animals, Diets, and Treatment

**Figure 4:**
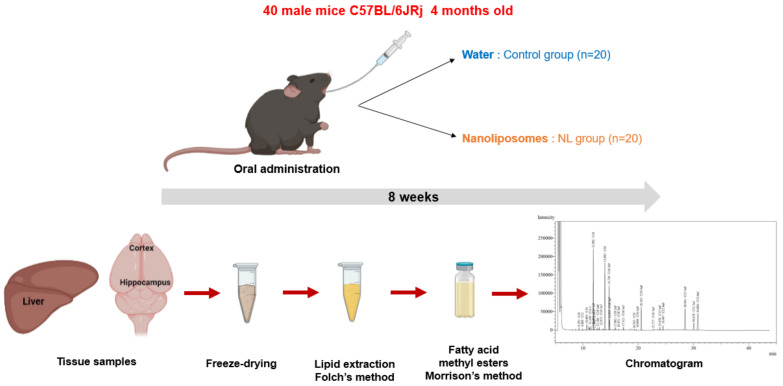
In Vivo NL treatment of mice: The experimental study involved 8 weeks (5 days/week) of oral administration of water or nanoliposomes to C57BL/6JRj mice. Lipid extraction from cerebral (cortex and hippocampus) and peripheral (liver) tissues, after freeze-drying, using the Folch method, followed by methylation of fatty acids and analysis of the composition of fatty acids via gas chromatography.

Male C57BL/6JRj mice (Janvier Labs, Le Genest-Saint-Isle) aged 4 months were used for this study (Figure 4). A group of 5 mice receiving no treatment (NT) was euthanized at T0. Two groups of 20 mice received either 250 µL of water (control group, C) or 12 mg of NLs in 250 µL (NL group), by gavage. Gavages were administered 5 days per week for 8 weeks. This procedure was performed using a force-feeding probe (curved probe (30°), 24 mm long, with a rounded end). Before the study and during the 8 weeks, submandibular blood samples were taken for the measurement of plasma lipid levels after a 3 h fast. At the end of the experimental period, the mice were euthanized using isoflurane, and the tissues were recovered (cortex, hippocampus, liver) and snap-frozen in liquid N(g).

Animals were housed in a certified animal facility, with constant temperature and humidity and an automatically controlled photoperiod (12 h light/12 h darkness). A standard chow diet (Harlan 2016 global rodent diet) and water were provided ad libitum. The assignment of animals to each group was randomized upstream so as to not place all of the mice from the same treatment in the same cage. The mice were fed daily with food (5–7 g, standard food, 2016 global rodent diet, Harlan-Envigo, Gannat, France) and water ad libitum. Animals were fasted 3 h before blood sampling. Gas chromatography analysis of the FA profile of the animal diet showed that it was composed of 60.4% PUFAs (specifically n-6 with 56.35 ± 0.07% linoleic acid), 21.2% MUFAs (20.27 ± 0.13% oleic acid), and 18.4% SFAs (15.52 ± 0.11% palmitic acid) (data not shown). There were no detectable levels of DHA or EPA.

Animal handling and experimental protocols were authorized in accordance with the European Communities Council Directive (EU 2010/63) for the use and care of laboratory animals, and in conformity with PHS policy on the Humane Care and Use of Laboratory Animals, incorporated in the Institute for Laboratory Animal Research (ILAR) Guide for Care and Use of Laboratory Animals. Throughout the study, the animals were observed on a daily basis to identify any possible changes in behavior so as to analyze the effects of treatment. Physiological and behavioral parameters were analyzed daily—in particular, eating, drinking, motor activities, urination, defecation, aggressiveness, vocalization, grooming, spasms, eyes closed outside of sleep, breathing, and appearance of the skin and hair.

The experimental protocol used was approved by the institutional board of ethics for animal experimentation (authorization N° 17198-2018092811144242 v3) on 28 January 2019.

#### 4.8.2. Analyses of Plasma Lipids

Blood samples were collected in EDTA-containing tubes, and centrifuged at 3000 rpm for 5 min to obtain plasma, which was stored at −20 °C for analysis. Plasma total cholesterol and triglycerides were determined using enzymatic kits (Total cholesterol, Wako (LabAssay); triglycerides (Sigma)) according to the manufacturers’ instructions.

#### 4.8.3. Tissue Samples

For tissue sampling, animals were anesthetized with 4% isoflurane, followed by decapitation to isolate the cortex and hippocampus. The livers were dissected, and all tissues were snap-frozen in liquid N_2_ and stored at −80 °C for analysis.

Then, the tissues were freeze-dried to remove water content that could have disturbed polar lipid extraction. After the freeze-drying, lipids were extracted using the Folch method (Figure 4) [76]. Following removal of the lipids in the chloroform/methanol phase into pre-weighed tubes, nonadecanoic acid was added as an internal control. The solvent was evaporated under nitrogen, and tubes were weighed to obtain the weight of lipids recovered for each tissue.

### 4.9. Statistical Analyses

Results were expressed as means ± SD. Results were analyzed using Student’s *t*-test to compare differences in each parameter measured between the control and nanoliposome groups at each timepoint separately for the cell culture and in vivo studies. One-way analysis of variance (ANOVA) followed by Tukey’s comparison test was used for total lipid mass measured at week 0 and at week 8, where lipid mass at week 0 was compared with that of week 8 in the two groups, and lipid mass of week 8 for the control and nanoliposome groups were compared. Statistical significance was considered as *p* < 0.05.

## 5. Conclusions

This study demonstrates for the first time PUFA NLs’ bioavailability in neuron cell culture and murine models. Interestingly, n-3 PUFA enrichment following NL administration was higher in the brain than in the liver, without weight gain or hyperlipidemia, with the highest levels in the cortex compared to the hippocampus; both regions participate in learning, as well as short- and long-term memory. By virtue of the origin and green extraction methods used to obtain salmon lecithin, these NLs are also low in toxicity, and biocompatible in vivo. We would propose that salmon lecithin NLs can be used as supplements for the delivery of n-3-rich PUFAs to the brain, thereby maintaining neuron function and synaptic plasticity, preventing age-related cognitive deficits, and reducing the risk of neurodegenerative diseases such as AD. This opens new possibilities in the development of strategies for the prevention and treatment of neurodegenerative diseases such as AD.

## Figures and Tables

**Table 1:** Fatty acid composition of salmon lecithin.

Fatty Acids (% Total)	Salmon Lecithin
Myristic acid (C14:0)	1.67 ± 0.02
Pentadecanoic acid (C15:0)	0.42 ± 0.01
Palmitic acid (C16:0)	20.08 ± 0.11
Heptadecanoic acid (C17:0)	0.41 ± 0.03
Stearic acid (C18:0)	4.98 ± 0.04
Saturated fatty acids	27.56
Palmitoleic acid (C16:1n-7)	1.74 ± 0.01
Oleic acid (C18:1n-9)	26.16 ± 0.21
Vaccenic acid (C18:1n-7)	2.56 ± 0.04
Eicosenoic acid (C20:1n-9)	0.63 ± 0.02
Monounsaturated fatty acids	31.09
Linoleic acid (C18:2n-6)	6.98 ± 0.11
Gamma-linolenic acid (C18:3n-6)	3.13 ± 0.05
Arachidonic acid (C20:4n-6)	2.11 ± 0.03
n-6	12.22
Alpha-linolenic acid (C18:3n-3)	1.63 ± 0.07
Eicosapentaenoic acid (C20:5n-3)	7.55 ± 0.05
Docosapentaenoic acid (C22:5n-3)	1.91 ± 0.08
Docosahexaenoic acid (C22:6n-3)	18.04 ± 0.09
n-3	29.13
Polyunsaturated fatty acids	41.35
DHA/EPA	2.39
n-6/n-3	0.42

The values are expressed as the mean ± standard deviation (SD) of % total fatty acids of triplicate determinations. DHA: docosahexaenoic; EPA: eicosapentaenoic.

**Table 2:** Fatty acid composition in primary cultures of embryo cortical neurons treated for 24 h, 48 h and 72 h with nanoliposomes (NL) and in the control group (C). The values are expressed as the mean ± SD. Statistical differences between groups for each fatty acid are shown at each incubation time (* *p* < 0.05, ** *p* < 0.01).

	24 h		48 h		72 h	
Fatty Acids (% Total)	C	NL	C	NL	C	NL
C14:0	3.11 ± 0.19	3.21 ± 0.31	3.22 ± 0.09	3.16 ± 0.06	3.24 ± 0.05	3.35 ± 0.09
C16:0	26.73 ± 0.40	27.21 ± 0.58	26.76 ± 0.27	28.44 ± 0.43	26.40 ± 0.56	27.76 ± 0.02 *
C17:0	2.75 ± 0.13	2.74 ± 0.11	2.70 ± 0.01	2.43 ± 0.22	2.55 ± 0.01	2.59 ± 0.08
C18:0	17.26 ± 0.81	16.82 ± 0.04	16.36 ± 0.67	16.93 ± 0.10	15.64 ± 0.78	16.55 ± 0.30
C20:0	2.86 ± 0.49	3.04 ± 0.02	3.14 ± 0.32	3.29 ± 0.05	3.99 ± 0.07	4.12 ± 0.27
Saturated	52.71 ± 0.66	53.02 ± 0.76	52.18 ± 1.34	54.25 ± 0.74	51.82 ± 1.22	54.37 ± 0.11
C16.1	4.32 ± 0.51	4.50 ± 0.26	4.95 ± 0.07	4.76 ± 0.28	5.65 ± 0.08	5.92 ± 0.05
C18:1n9	30.54 ± 0.47	29.83 ± 1.49	31.83 ± 0.79	29.46 ± 1.40	31.92 ± 0.81	28.02 ± 0.47 *
C18:1n7	3.18 ± 1.02	3.94 ± 0.13	3.04 ± 0.78	3.38 ± 0.34	3.26 ± 0.16	3.67 ± 0.02
C20:1n9	1.11 ± 0.03	1.22 ± 0.04	1.26 ± 0.05	1.02 ± 0.15	1.14 ± 0.03	1.08 ± 0.01
Monounsaturated	39.15 ± 2.03	39.49 ± 1.14	41.08 ± 1.55	38.62 ± 1.32	41.97 ± 0.85	38.69 ± 0.43 *
C18:2n6	2.27 ± 0.84	1.80 ± 0.01	1.75 ± 0.08	1.75 ± 0.17	1.63 ± 0.04	1.61 ± 0.06
C20:4n6	2.76 ± 0.09	2.46 ± 0.25	2.02 ± 0.09	2.16 ± 0.10	1.51 ± 0.24	2.10 ± 0.03
C20:3n6	1.69 ± 0.40	1.89 ± 0.01	1.86 ± 0.02	1.72 ± 0.24	1.87 ± 0.07	1.56 ± 0.23
n-6	6.72 ± 1.34	6.15 ± 0.26	5.63 ± 0.15	5.63 ± 0.51	5.01 ± 0.28	5.27 ± 0.26
C20:5n3	0.54 ± 0.05	0.59 ± 0.03	0.59 ± 0.03	0.79 ± 0.02 *	0.75 ± 0.08	0.99 ± 0.07
C22:6n3	0.88 ± 0.08	0.75 ± 0.15	0.52 ± 0.09	0.71 ± 0.05	0.45 ± 0.01	0.68 ± 0.01 **
n-3	1.42 ± 0.04	1.34 ± 0.12	1.11 ± 0.06	1.50 ± 0.07 *	1.20 ± 0.09	1.67 ± 0.06 *
Polyunsaturated	8.14 ± 1.37	7.49 ± 0.38	6.74 ± 0.21	7.13 ± 0.58	6.21 ± 0.36	6.94 ± 0.32

**Table 3:** Fatty acid composition in the cortex, hippocampus, and liver of mice after 8-week NL treatment. The values are expressed as the mean ± SD. Statistical differences between the control and nanoliposome groups for each fatty acid are shown for each tissue (* *p* < 0.05, ** *p* < 0.01, *** *p* < 0.001).

	Cortex		Hippocampus		Liver	
Fatty Acids (mg/g Dry Tissue)	C	NL	C	NL	C	NL
C10:0	0.18 ± 0.04	0.44 ± 0.06 *	0.44 ± 0.19	0.35 ± 0.05	-	-
C12:0	0.10 ± 0.02	0.25 ± 0.04 *	0.19 ± 0.03	0.22 ± 0.04	-	-
C14:0	0.45 ± 0.10	1.09 ± 0.12 **	0.61 ± 0.05	0.90 ± 0.19	0.16 ± 0.01	0.14 ± 0.03
C16:0	6.16 ± 1.18	16.30 ± 2.70 *	12.99 ± 1.91	14.61 ± 3.27	12.52 ± 1.56	10.24 ± 2.14
C18:0	6.03 ± 1.17	15.58 ± 1.92 **	12.75 ± 1.71	14.31 ± 2.65	5.53 ± 0.67	4.87 ± 1.01
C20:0	0.08 ± 0.01	0.25 ± 0.01 ***	0.17 ± 0.01	0.23 ± 0.07	0.29 ± 0.03	0.28 ± 0.07
Saturated	13.00 ± 2.51	33.91 ± 4.82 **	27.15 ± 3.73	30.62 ± 6.27	18.50 ± 2.27	15.53 ± 3.14
C14:1	0.36 ± 0.08	0.78 ± 0.05 **	0.33 ± 0.16	0.46 ± 0.08	-	-
C14:1n-9	0.29 ± 0.06	0.68 ± 0.07 **	0.29 ± 0.25	0.58 ± 0.10	-	-
C16:1	0.10 ± 0.01	0.36 ± 0.02 ***	0.49 ± 0.21	0.39 ± 0.13	-	-
C16:1n-9	0.14 ± 0.02	0.44 ± 0.14	0.34 ± 0.04	0.44 ± 0.18	0.16 ± 0.01	0.15 ± 0.04
C16:1n-7	0.23 ± 0.05	0.66 ± 0.06 **	0.21 ± 0.11	0.44 ± 0.08	0.99 ± 0.12	0.85 ± 0.27
C18:1n-9	3.94 ± 0.93	10.45 ± 2.10 *	8.09 ± 0.98	10.19 ± 2.68	5.76 ± 0.08	4.98 ± 1.17
C18:1n-7	0.88 ± 0.19	2.03 ± 0.54	1.53 ± 0.13	1.88 ± 0.52	1.13 ± 0.03	0.95 ± 0.19
C20:1n-9	0.07 ± 0.02	0.28 ± 0.02 **	0.14 ± 0.01	0.28 ± 0.11	0.69 ± 0.16	0.56 ± 0.16
C22:1n-9	0.40 ± 0.12	1.01 ± 0.11 **	0.72 ± 0.11	0.88 ± 0.19	-	-
Monounsaturated	6.41 ± 1.45	16.69 ± 2.71 *	12.14 ± 0.96	15.54 ± 3.92	8.73 ± 0.40	7.49 ± 1.80
C18:2n-6	0.45 ± 0.11	1.20 ± 0.56	1.21 ± 0.17	1.71 ± 0.82	6.34 ± 0.67	5.66 ± 1.06
C18:3n-6	0.07 ± 0.01	0.18 ± 0.01 **	0.20 ± 0.04	0.22 ± 0.05	0.09 ± 0.02	0.07 ± 0.02
C20:4n-6	2.58 ± 0.51	6.59 ± 0.80 **	4.51 ± 0.05	5.89 ± 1.48	6.18 ± 0.25	4.90 ± 0.97
C22:4n-6	0.64 ± 0.14	1.61 ± 0.11 **	1.03 ± 0.09	1.34 ± 0.28	0.23 ± 0.02	0.20 ± 0.07
C22:5n-6	3.81 ± 0.72	10.67 ± 1.64 *	5.62 ± 0.43	7.65 ± 1.48	3.70 ± 1.19	3.63 ± 0.81
n-6	7.55 ± 1.48	20.25 ± 3.05 *	12.57 ± 0.78	16.81 ± 4.12	16.54 ± 2.15	14.46 ± 2.86
C18:3n-3	0.28 ± 0.08	0.72 ± 0.03 **	0.47 ± 0.07	0.53 ± 0.14	0.26 ± 0.01	0.26 ± 0.05
C20:5n-3	0.14 ± 0.05	0.33 ± 0.03 *	0.28 ± 0.08	0.33 ± 0.08	0.21 ± 0.03	0.20 ± 0.05
C22:5n-3	1.60 ± 0.38	4.21 ± 0.37 **	2.78 ± 0.22	3.60 ± 0.78	0.56 ± 0.08	0.52 ± 0.11
C22:6n-3	3.85 ± 0.92	10.20 ± 0.91 **	6.68 ± 0.53	8.66 ± 1.91	1.35 ± 0.20	1.26 ± 0.26
n-3	5.87 ± 1.42	15.47 ± 1.22 **	10.21 ± 0.89	13.12 ± 2.91	2.38 ± 0.31	2.24 ± 0.47
Polyunsaturated	13.42 ± 2.91	35.71 ± 4.26 **	22.78 ± 1.67	29.93 ± 7.03	18.92 ± 2.47	16.70 ± 3.32
n-6/n-3	1.29 ± 0.06	1.30 ± 0.10	1.23 ± 0.03	1.28 ± 0.03	6.99 ± 0.02	6.49 ± 0.25
AA/DHA	0.67 ± 0.03	0.64 ± 0.02	0.68 ± 0.05	0.68 ± 0.02	4.64 ± 0.52	3.93 ± 0.34
PUFA/SFA	1.03 ± 0.02	1.06 ± 0.04	0.84 ± 0.05	0.97 ± 0.03	1.02 ± 0.01	1.08 ± 0.07
MUFA/SFA	0.49 ± 0.02	0.49 ± 0.05	0.45 ± 0.03	0.50 ± 0.02	0.47 ± 0.04	0.48 ± 0.07

**Table 4:** Total lipid mass (mg/g dry tissue) in the cortex, hippocampus, and liver of mice before (week 0) and after 8-week nanoliposome treatment. The values are expressed as the mean ± SD. Statistical differences (*p* < 0.05) are shown for each tissue; week 0 is compared with week 8 for the control (C) group ^a^ and week 8 for the nanoliposome (NL) group ^b^, and for week 8 the C group is compared with the NL group ^c^. Δ % represents the percentage of change.

		Cortex			Hippocampus			Liver		
		Week 0	Week 8	Δ %	Week 0	Week 8	Δ %	Week 0	Week 8	Δ %
**Lipids**	**C**	0.18 ± 0.11	0.36 ± 0.20	97%	1.54 ± 1.36	3.54 ± 1.38	130%	0.02 ± 0.01	0.31 ± 0.02 ^a^	1534%
(**mg**/**g dry tissue**)	**NL**		0.42 ± 0.09 ^b^	132%		4.18 ± 1.89	171%		0.15 ± 0.03 ^b,c^	665%

## Data Availability

Not applicable.
